# MR‐safe personal radiation dosimeters

**DOI:** 10.1002/acm2.12115

**Published:** 2017-06-06

**Authors:** E. Tchistiakova, A. Kim, W. Y. Song, G. Pang

**Affiliations:** ^1^ Sunnybrook Health Sciences Centre/Odette Cancer Centre Toronto ON Canada; ^2^ Faculty of Medicine Department of Radiation Oncology University of Toronto Toronto ON Canada

**Keywords:** MRI‐guided radiation therapy, MR‐safe radiation detectors, radiation protection

## Abstract

Magnetic resonance imaging **(** MRI) is being rapidly integrated for cancer treatments—such systems are referred to as MRI‐guided radiation therapy (MRIgRT). As the magnet of an MRI scanner is always on, the presence of a strong static magnetic field from the MRI scanner during radiotherapy delivery presents new challenges. One of the challenges is that a personal radiation dosimeter used to estimate the radiation dose deposited in an individual wearing the device must be MR‐safe. No such devices, however, are currently available. In this work we first modified an existing personal dosimeter (by removing a metal clip) to make it MR‐safe and then investigated potential effects of magnetic field on dosimeter readings, i.e., optically stimulated luminescent dosimeter (OSLD) readings. We found that the effect of magnetic field on OSLD sensitivity was within radiation protection tolerance levels. OSLD personal dosimeters can be directly used in conjunction with MRIgRT radiation protection purposes.

## 1 INTRODUCTION

Integration of magnetic resonance imaging **(**MRI) for cancer treatments is on the rise. MR‐Linac and MRI‐guided brachytherapy, which combine MRI scanner with radiation treatment delivery systems, are two examples of this new development.^1^, ^2^, ^3^, ^4^, ^5^ These hybrid systems are collectively referred to as MRI‐guided radiation therapy (MRIgRT) systems. Compared to other imaging modalities, MRI provides superior soft‐tissue contrast, which is needed for high accuracy radiotherapy. Along with the benefits of superior contrast, MRI also presents new challenges. One such challenge is a constant exposure of radiotherapy equipment to a strong static magnetic field from an MRI scanner, which is always on even when no images are being acquired.

Personal radiation dosimeters are used to estimate the radiation dose deposited in an individual wearing the device. These dosimeters must be MR‐safe to be used in the radiation facilities that incorporate MRI.

Currently, there are two main types of personal dosimeters that are used in cancer centers: one is based on thermoluminescent dosimetry (TLDs) and the other on optically stimulated luminescent dosimetry (OSLDs).^6^, ^7^ The badge typically contains multiple TLD or OSLD chips, each under a different filter to simulate various depths in tissue.

We propose that a slightly modified version of these radiation badges may be used for radiation protection dosimetry in MRI zones used for radiation therapy. The small modification is to eliminate the metal clips in standard radiation badges. The modified badges could be placed in plastic pouches with lanyards for easy wearing.

The objective of this study was to determine if a modified standard radiation badge can be used as a personal dosimeter in MRIgRT suites. To this end, the potential effects of magnetic field on the sensitivity of TLDs and OSLDs need to be investigated. Previous research by Mathis et al^8^ investigated the effect of magnetic field on the sensitivities of selected radiation dosimeters including TLDs and OSLDs. The doses delivered to the dosimeters in that study, however, were ≥2 Gy, which is much higher than the typical doses (<1 cGy or 10 mSv) seen in radiation protection dosimetry. In this work, we investigated potential magnetic effects on the sensitivity of OSLDs that are located inside a radiation badge in the dose range applicable to radiation protection for both high‐energy (megavoltage [MV]) x‐rays and low‐energy (kilovoltage [kV]) *γ* rays. We conducted two sets of experiments. The first is to test the OSLD sensitivity for sequential exposure to MV radiation and high strength static magnetic field; the second is to test the OSLD sensitivity to simultaneous exposure to kV *γ* radiation and a high strength static magnetic field.

## 2 METHOD AND MATERIALS

### 2.A Modification of current personal dosimeters

**Figure 1:**
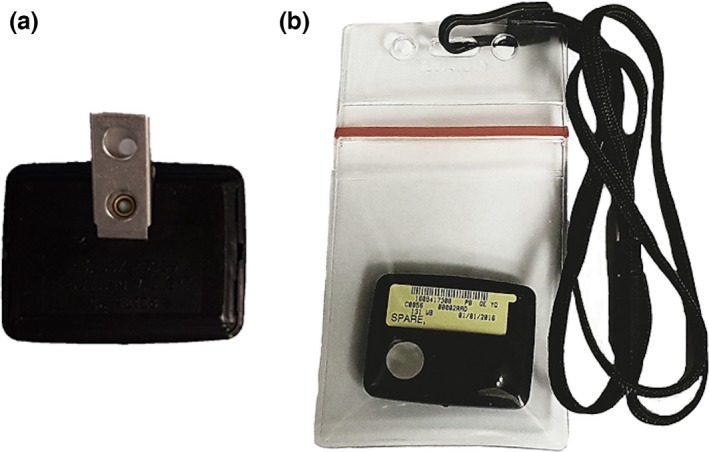
(a) Back of a standard radiation protection badge (Mirion Technologies Inc., Irvine, USA); (b) modified version stripped of the metal holder clip.

The metal holder clip in a standard radiation badge (Mirion Technologies Inc., Irvine, USA) was removed to make it MR‐safe (Fig. 1). The badge was tested within a 3T Philips Achieva MRI scanner. A ferromagnetic detector (Safescan Target Scanner, Mednovus) was also used to confirm that the modified badge does not contain any ferromagnetic components. To allow for the in‐house repeated measurements, four Al_2_O_3_ nanoDots OSLDs (Landauer, Glenwood, Illinois, USA) were placed inside the radiation badge. OSLDs were chosen to allow for repeated measurements to be obtained throughout the course of several months. Al_2_O_3_:C material is well suited for personal dosimetry^9^ and has been previously used in the Luxel+ dosimeters (Landauer). An in‐house build frame was used to fit the nanoDots OSLDs in the standard badge frame. The filters used in front of the four OSLDs were exactly the same as those in the standard radiation badge and consist of combinations of Cu, teflon, mylar, and plastic materials for a total thickness of 445, 1100, 57, 412 mg cm^2^, respectively. All badges used in subsequent experiments were modified in the same way.

A commercially available optically stimulated luminescence dosimetry system, the MicroStar reader from Landauer (Glenwood, Illinois, USA), was used for dosimeter readings. In our experiments, all nanoDots were exposed to dose levels classified as “low” by the software and were therefore readout in “Low dose” mode.

### 2.B Measurement of the magnetic field effect on OSLD sensitivity

#### 2.B.1 Sequential exposure to MV radiation and magnetic field

In the first set of experiments, a modified OSLD badge was first exposed to radiation in the absence of magnetic field. The irradiation was conducted using a 6 MV Synergy MLCi unit (Elekta Limited, West Sussex, UK). The field size was set to 24 cm × 24 cm and 1000 MUs were delivered. An ion chamber survey meter (Model 451B, Fluke Corporation, Everett, USA) was used to locate the position within the bunker where the equivalent dose is approximately 1 mSv. The OSLD badge was then placed in the determined location and irradiated. To determine the exact dose received by the badge, the OSLD measurements were taken before and after the irradiation using the MicroStar reader, averaging across five measurements. The irradiated badge and a secondary, nonirradiated OSLD badge were then placed in a 3T Philips Achieva scanner room within the 0.2 T line (i.e., taped at the back of the bore). A third, “Control” badge was placed outside the MRI room in the patient preparation area. A baseline measurement for OSLDs in the control and the nonirradiated badges were obtained prior to installation, similarly to the irradiated badge. All three badges were read weekly for 3 months (standard time for radiation badge cycle) using MicroStar InLight Reader. No radiation exposure was delivered to any of the badges during the 3 months except for that from the natural background radiation. Following the 3‐month period, the “MRI+ Irradiation” and the “Control” badges received an additional radiation dose of 1 mSv to compare the sensitivity of OSLDs to radiation post magnetic field exposure.

Finally, one of the OSLDs from the irradiated badge was read off consecutively 65 times to establish the amount of dose discharge due to effect of repeated readings alone. The number of readings was approximately equivalent to that performed during the 3‐month period (13 weeks × 5 readings).

#### 2.B.2 Simultaneous exposure to kV γ radiation and magnetic field

**Figure 2:**
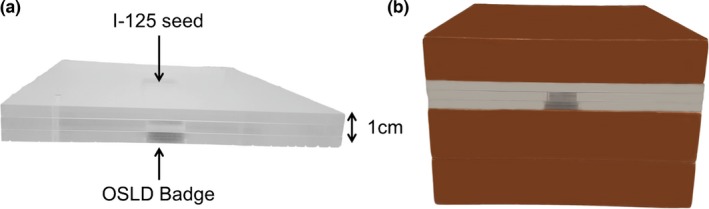
Setup for I‐125 experiment. (a) In‐house manufactured jig designed to reproducibly set up the badge 1 cm away from I‐125 seed and (b) jig setup between blocks of solid water for extra shielding.

In the second set of experiments, I‐125 radioactive loose seeds (IsoAid, Port Richey, USA) with 0.4 mCi each were ordered. A modified OSLD badge was placed in a holder about 1 cm below the radioactive seed (Fig. 2) for an hour (with calculated exposure of ~0.54 cGy). The same experiment was repeated inside the 3T MRI scanner so that the OSLDs were exposed to both, radiation and magnetic field (with calculated exposure of ~0.49 cGy). OSLD measurements were made before and after the exposure, averaging across five readings. OSLD readings obtained with and without the magnetic field were compared.

## 3 RESULTS

### 3.A Sequential exposure to MV radiation and magnetic field

**Figure 3:**
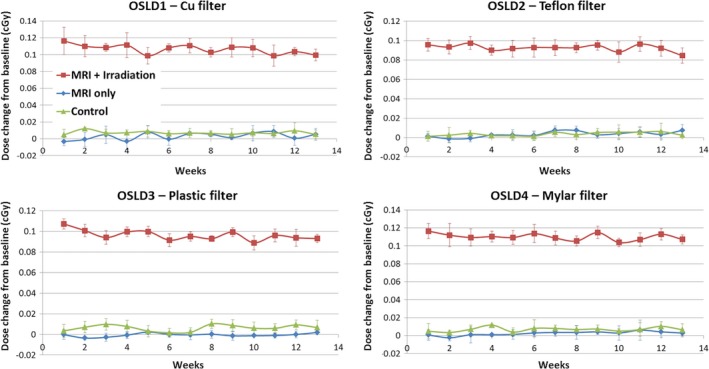
Dose change from baseline in OSLD measurements over 3 months. Values are corrected for baseline measurements at Week 0 (before the irradiation of “MRI+ Irradiation” badge at the beginning of the first experiment). Measurements are mean ± standard deviation of the five readings obtained at each time point.

Results from the first set of experiments during the 3‐month period are shown in Fig. 3. These results suggest that exposure of OSLDs to the magnetic field post irradiation has minimal additional effect on OSLD measurements (the slight signal decrease over time observed in the “MRI+ Irradiation” badge is likely due to fading. More details are given in the Discussion section).

We note here that signal depletion due to partial discharge over five readings (number of measurements per week) was measured to be 0.003 cGy or 0.75%. Data shown in Fig. 3 were not corrected for fading, partial discharge over multiple readings or natural background radiation (see 5 section for details).

After 3‐month long exposure to magnetic field, radiation sensitivity of OSLDs was found to be within 5.2 ± 2.4% of the control badge. This difference is consistent with expected OSLD‐to‐OSLD variability and is within the tolerance levels for radiation protection purposes (~10%).^9^, ^10^

### 3.B Simultaneous exposure to kV *γ* radiation and magnetic field

**Figure 4:**
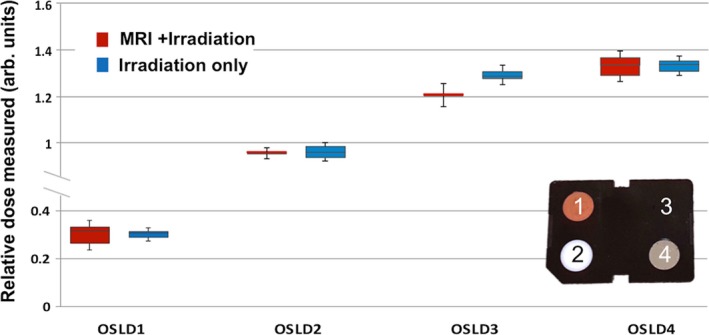
OSLD measurement after exposure to radiation (from I‐125) with and without magnetic field (error bars represent the standard deviation for five repeated OSLD measurements). The dose was normalized to the calculated exposure dose of 0.49 cGy and 0.54 cGy for experiment with and without magnetic field, respectively. The percent difference between Irradiation only and MRI+ Irradiation were −0.3%, −0.5%, 7.3%, and 0.7% for OSLDs 1–4, respectively. Also shown are the filters of the dosimetry badge used to estimate the dose to various tissue depths.

Simultaneous exposure of OSLDs to kV *γ* radiation and magnetic field showed no significant difference compared to exposure to radiation alone (Fig. 4). OSLD no. 3 showed greater difference between measurements with and without magnetic field; however, the difference was still within one standard deviation of the five readings. Variation of measured dose from delivered dose and between OSLDs were attributed to the different filters in the badge used to estimate dose to different tissue depths and small inherent OSLD‐to‐OSLD differences.

## 4 DISCUSSION

Exposure of sensitive radiation dosimetry equipment to strong magnetic field of MR is an important aspect to consider as MRIgRT is being developed. One concern with simultaneous exposure to magnetic field and radiation is the electron return effect (ERE).^11^ ERE refers to the increase in the surface dose due to the secondary electrons that are forced back into the tissue by the Lorentz force. New treatment planning systems are being developed that will allow to account for these effects during MRIgRT. The impact of ERE on the dosimetric equipment, however, is still not well understood. Furthermore, it is also unclear if a sequential exposure to radiation and magnetic field may impact dosimetric accuracy.

The results of this study demonstrate that both sequential and simultaneous exposure of OSLDs to radiation and magnetic field had minimal (<8%) effect on radiation sensitivity of OSLDs and their ability to hold radiation‐induced charge for an extended period of time in a strong magnetic field. These results are consistent with the findings reported by Mathis et al^8^ and are within the expected OSLD‐to‐OSLD variability and the tolerance levels for radiation protection purposes (~10%).^9^, ^10^

Charge depletion due to the repeated OSLD readouts each weak was of the same magnitude as the natural background radiation received by the OSLD over 1 week (~0.2*μ*Sv/h*24 h*7 days = 0.034 mSv). As the two contributions have an opposite effect, they canceled each other out and, therefore, were not corrected for in Fig. 3. The signal decrease over time observed in the “MRI+ Irradiation” badge measurements was 0.0006 cGy/week (based on the linear model fit), for a total of 4.0% decrease after 13 weeks. This value is comparable to that due to fading reported by Dunn et al.^12^ These authors also demonstrated that the decline due to fading levels off after the first couple of weeks post irradiation. This may explain why no significant fading was observed in the “Control” and “MRI only” OSLDs that were not irradiated at the beginning of the first experiment.

The goal of the earlier studies that examined combined effects of radiation and magnetic field on OSLDs was their validation for quality assurance and *in vivo* dosimetry purposes.^8^ In the current study, our goal was to evaluate OSLDs from a radiation protection perspective. This inherent contrast in primary objectives of the two studies is reflected in several experimental differences. Firstly, the OSLD measurements in the current experiment were taken over a longer period of time (3 months), to examine potential effects of magnetic field not only on radiation sensitivity but also the ability of OSLDs to hold radiation‐induced charge in a strong magnetic field for a period of standard radiation badge cycle. Secondly, the dose that the OSLD badge was exposed to in this experiment was much lower (~1 mSv) than in the previous study (>2 Gy), which is a better representation of the dose typically seen in radiation protection dosimetry. Finally, in the current experiment, we examined two types of radiation sources, Linacs and radioactive seeds, as both of these applications will benefit from MRI guidance (i.e., MR‐Linac and MRI‐guided brachytherapy) and the development of which is currently ongoing.

In this study, we examined two scenarios of potential exposure of personal dosimetry badge to radiation and magnetic field in the clinic. There are, however, several limitations that need to be mentioned. First, simultaneous exposure was performed with radioactive seeds only. Although this scenario is valid for MR‐guided brachytherapy, exposure to MV energies representative of external beam therapy (i.e., MRI‐Linac) will need to be addressed as the technology becomes available. Second, in this study, we focused on potential effects of a magnetic field on irradiated OSLD detectors. Although previous research by Mathias et al. showed no effect of magnetic field on TLD sensitivity to high‐dose radiation exposure,^8^ its sensitivity to lower doses, characteristic to radiation protection dosimetry, still needs to be confirmed. We also chose not to include “Irradiation only” scenario in our study as its effects on OSLDs has been well documented previously by Jursinic et al.^7^ and Lee et al.^9^ Finally, the radiation protection badges must be worn such that the filters within the badge are facing the radiation source to properly estimate the dose to different tissue depths. Care must be taken to ensure that the badge is not flipped if used with a lanyard. Alternatively, a plastic clip could be used to secure the pouch with a badge.

## 5 CONCLUSION

We investigated the use of a slightly modified OSLD personal dosimeter in MR suite for MRIgRT. We found that the modified OSLD personal dosimeter is MR‐safe and the effect of magnetic field on OSLD readings is negligible. This work provides a technical solution for clinical centers that are seeking MR‐compatible personal radiation dosimeters.

## ACKNOWLEDGMENTS

We thank Mr. Harry Easton of Odette Cancer Centre for providing engineering support and Dr. Alyaa Elzibak of Odette Cancer Centre for helpful discussions.

## CONFLICT OF INTEREST

The authors have no conflict of interest to disclose.
